# Bioactive Compounds from the Red Sea Marine Sponge *Hyrtios* Species

**DOI:** 10.3390/md11041061

**Published:** 2013-03-28

**Authors:** Diaa T. A. Youssef, Lamiaa A. Shaala, Hani Z. Asfour

**Affiliations:** 1 Department of Natural Products, Faculty of Pharmacy, King Abdulaziz University, Jeddah 21589, Kingdom of Saudi Arabia; 2 Natural Products Unit, King Fahd Medical Research Center, King Abdulaziz University, Jeddah 21589, Kingdom of Saudi Arabia; E-Mail: lshalla@kau.edu.sa; 3 Department of Medical Parasitology, Faculty of Medicine, Princess Al-Jawhara Center of Excellence in Research of Hereditary Disorders, King Abdulaziz University, Jeddah 21589, Kingdom of Saudi Arabia; E-Mail: hasfour@hotmail.com

**Keywords:** Red Sea sponge, *Hyrtios* species, alkaloids, antimicrobial activity, free radical scavenging activity, cancer growth inhibition activity

## Abstract

In continuation of our search for drug leads from Red Sea sponges we have investigated the ethyl acetate fraction of the organic extract of the Red Sea sponge *Hyrtios* species. Bioassay-directed fractionation of the active fraction resulted into the identification of three new alkaloids, hyrtioerectines D–F (**1**–**3**). Hyrtioerectines D–F belong to the rare marine alkaloids in which the indole and β-carboline fragments of the molecule are linked through C-3/C-3 of both moieties. The structures of the isolated compounds were established based on different spectroscopic data including UV, IR, 1D and 2D NMR (COSY, HSQC, and HMBC) and high-resolution mass spectral studies. The antimicrobial activity against several pathogens and the free radical scavenging activity of the compounds using DPPH reagent were evaluated. In addition, the growth inhibitory activity of the compounds against three cancer cell lines was also evaluated. Hyrtioerectines D–F (**1**–**3**) displayed variable antimicrobial, free radical scavenging and cancer growth inhibition activities. Generally, compounds **1** and **3** were more active than compound **2**.

## 1. Introduction

Sponges belonging to the genus *Hyrtios* (Demospongiae class, Dictyoceratida order, Thorectidae family) [1] have proven to be a rich source of biologically active diverse secondary metabolites of different classes. Prominent reported classes from this genus include sesterterpenes [2,3,4,5,6], sesquiterpenes [7,8,9], macrolides [10,11], indole and β-carboline alkaloids [12,13,14,15,16]. Indole derivatives possess different biological activities including anticancer, antibiotic, anti-inflammatory and antioxidant activities [17,18]. Secondary metabolites of the genus *Hyrtios* displayed diverse biological activities [2,3,5,7,10,11,12,13,15,19]. In the course of our ongoing efforts to identify drug leads from Red Sea marine organisms, we investigated the ethyl acetate fraction of an organic extract of the Red Sea marine sponge *Hyrtios* species. 

**Figure 1:**
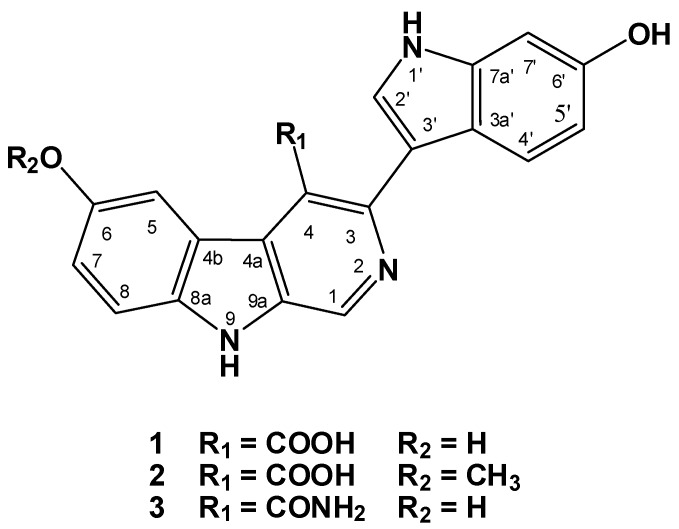
Structures of hyrtioerectines D–F (**1**–**3**) isolated from the marine sponge *Hyrtios* species.

Chromatographic fractionation and final HPLC purification of the active ethyl acetate fraction resulted into the isolation of three alkaloids, hyrtioerectines D–F (**1**–**3**) (Figure 1). The assignment of the structure of the compounds was based on one- and two-dimensional NMR studies including COSY, HSQC, and HMBC together with IR and high-resolution mass spectral studies.

## 2. Results and Discussion

### 2.1. Purification of Compounds **1–3**

Successive chromatographic fractionation of the antimicrobial ethyl acetate fraction of an organic extract of the Red Sea sponge *Hyrtios* species using a combination of liquid-liquid partition, size exclusion chromatography on Sephadex LH-20 and final HPLC purification of the active fraction on C18 HPLC column afforded three new alkaloids hyrtioerectines D–F (**1**–**3**). The structure determination of the compounds was assigned from one- and two-dimensional NMR and high-resolution mass spectral studies.

### 2.2. Structure Elucidation of Compounds **1–3**

**Figure 2:**
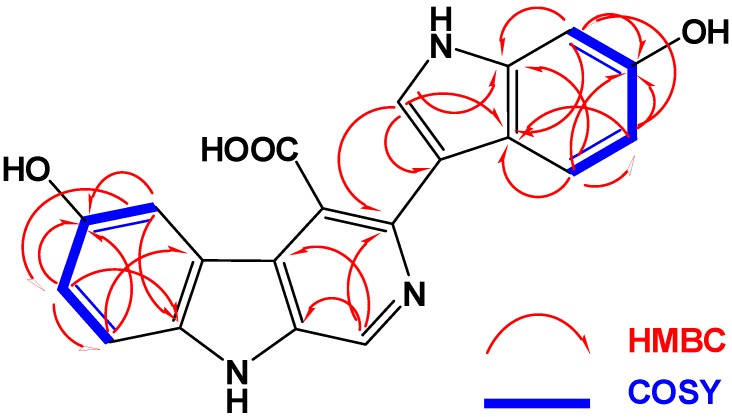
COSY and HMBC correlations observed for compound **1**.

The HRMS of compound **1** established a molecular formula C_20_H_13_N_3_O_4_ requiring 16 degrees of unsaturation. Compound **1** gave a bluish color on the TLC with FeCl_3_ indicating its phenolic nature. Its ^1^H NMR spectrum (in methanol-*d*_4_, Table 1) showed resonances for eight aromatic protons ranging from 6.80 to 9.64 ppm, suggesting the absence of any aliphatic moiety in the molecule. The ^13^C NMR spectrum of **1** (Table 1) displayed resonances for 20 carbons including 8 methines and 12 quaternary carbons. Two of the ^13^C quaternary resonances were deshielded at δ 154.3 (C-6) and 153.0 (C-6′) suggesting the oxygenation of their corresponding carbons. Interpretation of the 1D (^1^H and ^13^C) and 2D (^1^H–^1^H COSY, HSQC and HMBC) NMR spectroscopic data supported the presence of two ABX systems for trisubstituted β-carboline (H-5, H-7 and H-8) and disubstituted indole (H-4ʹ, H-5ʹ and H-7ʹ) moieties, respectively. These two moieties include 15 degrees of unsatuartion suggesting the need for an additional degree of unsaturation in **1**. Interpretation of the NMR data (^1^H, COSY and HSQC) supported the 3,4,6-trisubstitution of the β-carboline moiety. This was evident from the resonating signals at δ 8.07 (1H, d, *J* = 2.0, H-5), 6.80 (1H, dd, *J* = 8.5, 2.0, H-7), 7.30 (1H, d, *J* = 8.5, H-8) and 9.64 (s, H-1). Additional signals at δ 8.87 (1H, s, H-2ʹ), 7.57 (1H, d, *J* = 8.7, H-4ʹ), 7.13 (1H, dd, *J* = 8.7, 2.3, H-5ʹ) and 7.60 (1H, d, *J* = 2.3, H-7ʹ) were assigned as 3,6-disubstituted indole moiety. The HSQC experiment allowed the unambiguous assignment of the protonated methine carbons in **1** (Table 1). The quaternary carbons were unambiguously assigned from HMBC data (Table 2 and Figure 2). For example, the location of the OH moieties at C-6 and C-6′ was supported from HMBC correlations of H-5/C-6, H-7/C-6, H-8/C-6 and H-4′/C-6′, H-5′/C-6′ and H-7′/C-6′ (Table 2). The connection of the indole and β-carboline moieties through C-3 and C-3ʹ was supported from a ^3^*J*_CH_ HMBC cross peak between H-2ʹ of the indole moiety (δ 8.87) and C-3 of the β-carboline moiety (δ 138.1). Finally, the quaternary carbon resonating at δ 173.0 (C-8′) was assigned as a carboxylic moiety at C-4 completing the degrees of unsaturation in **1**. This was supported by an IR band at 1725 cm^−1^ for the carbonyl moiety of the carboxylic acid. Furthermore, the MS fragment ion at *m/z* 315.1009 with the molecular formula C_19_H_13_N_3_O_2_ [M − COOH + H]^+^ supported the presence of the carboxylic functionality, thus completing the assignment of **1**.

Compound **2**displayed a pseudomolecular ion peak at *m/z* 374.1138 corresponding to C_21_H_16_N_3_O_4_ [M + H]^+^, being 14 mass unit (CH_2_) more than **1** and requiring 16 degrees of unsaturation. Comparison of the NMR data of **2** with those of **1** showed identical similarity of the ^1^H and ^13^C NMR resonances of both compounds (Table 1). Moreover, a new three-proton singlet in ^1^H NMR at δ 3.84 (H_3_-9ʹ) correlates in the HSQC with the ^13^C NMR signal at δ 55.8 (C-9ʹ) suggesting the presence of a methoxyl moiety in **2** at C-6. The assignment of the methoxyl moiety at C-6 was secured from HMBC cross peak of OCH_3_/C-6 (Table 2). Additionally, the unambiguous assignments of the protonated and quaternary carbons in **2** were secured from HSQC and HMBC experiments, respectively, thus completing the assignment of **2**.

**Table 1:** NMR spectroscopic data for hyrtioerectines D–F (**1**–**3**) (in methanol-*d*_4_).

	Hyrtioerectine D		Hyrtioerectine E		Hyrtioerectine F	
Position	δ_C_ (mult.) ^a^	δ_H_, mult. (*J* in Hz)	δ_C_ (mult.) ^a^	δ_H_, mult. (*J* in Hz)	δ_C_ (mult.) ^a^	δ_H_, mult. (*J* in Hz)
1	140.8 (CH)	9.64, s	141.2 (CH)	9.66, s	139.6 (CH)	9.71, s
3	138.1 (qC)		138.2 (qC)		138.3 (qC)	
4	138.8 (qC)		138.8 (qC)		138.8 (qC)	
4a	115.9 (qC)		115.8 (qC)		115.9 (qC)	
4b	130.1 (qC)		130.3 (qC)		130.4 (qC)	
5	108.2 (CH)	8.07, d (2.0)	110.1 (CH)	8.10, d (2.2)	107.2 (CH)	8.09, d (2.2)
6	154.3 (qC)		156.2 (qC)		154.2 (qC)	
7	113.5 (CH)	6.80, dd (8.5, 2.0)	113.5 (CH)	6.78, dd (8.5, 2.2)	113.7 (CH)	6.76, dd (8.5, 2.2)
8	113.1 (CH)	7.30, d (8.5)	113.1 (CH)	7.27, d (8.5)	113.5 (CH)	7.32, d (8.5)
8a	132.1 (qC)		132.2 (qC)		132.4 (qC)	
9a	133.2 (qC)		133.1 (qC)		133.2 (qC)	
2′	119.6 (CH)	8.87, s	119.5 (CH)	8.88, s	119.6 (CH)	8.87, s
3′	123.2 (qC)		123.2 (qC)		123.2 (qC)	
3a′	137.5 (qC)		137.5 (qC)		137.5 (qC)	
4′	114.1 (CH)	7.57, d (8.7)	114.1 (CH)	7.61, d (8.7)	114.1 (CH)	7.64, d (8.0)
5′	119.7 (CH)	7.13, dd (8.7, 2.3)	119.6 (CH)	7.15, dd (8.7, 2.2)	119.7 (CH)	7.17, dd (8.7, 2.0)
6′	153.0 (qC)		153.1 (qC)		153.0 (qC)	
7′	106.7 (CH)	7.60, d (2.3)	106.9(CH)	7.62, d (2.2)	106.7 (CH)	7.65, d (2.0)
7a′	132.6 (qC)		132.5 (qC)		132.6 (qC)	
8′	173.0 (qC)		173.0 (qC)		164.2 (qC)	
9′			55.8 (CH_3_)	3.84, s		

^a^ multiplicities of the signals were obtained from HSQC experiments; qC = quaternary carbon, CH = methine; CH_3_ = methyl.

**Table 2:** Selected HMBC correlations for hyrtioerectines D–F (**1**–**3**) (in methanol-*d*_4_).

C#	Hyrtiooerectine D (1)	Hyrtioerectine E (2)	Hyrtioerectine F (3)
	HMBC (H→C#)	HMBC (H→C#)	HMBC (H→C#)
3	H-1, H-2′	H-1, H-2′	H-1, H-2′
4	-	-	-
4a	H-1, H-5	H-1	H-1, H-5
4b	H-8	H-8	H-8
5	H-7	H-7	H-7
6	H-5, H-7, H-8	H-5, H-7, H-8, H_3_-9′	H-5, H-8
7	H-5	H-5	H-5
8	H-7	H-7	H-7
8a	H-5, H-7	H-5, H-7	H-5, H-7
9a	H-1	H-1	H-1
2′	-	-	-
3′	H-2′	H-2′	H-2′
3a′	H-2′, H-4′, H-5′, H-7′	H-2′, H-4′, H-7′	H-2′, H-4′, H-7′
4′	H-5′	H-5′	H-5′
5′	H-4′, H-7′	H-4′, H-7′	H-4′, H-7′
6′	H-4′, H-5′, H-7′	H-4′, H-5′, H-7′	H-4′, H-5′, H-7′
7′	H-5′	H-5′	H-5′
7a′	H-2′, H-4′, H-7′	H-2′, H-4′, H-7′	H-2′, H-4′, H-7′

Compound **3** displayed a pseudomolecular ion peak at *m/z* 359.1142 corresponding to C_20_H_15_N_4_O_3_ [M + H]^+^, being one mass unit less than **1** and requiring the same degrees of unsaturation. Again, comparison of the NMR data of **3** with those of **1** showed close similarity of two compounds (Table 1). The presence of the amide moiety in **3** instead of the carboxylic moiety in **1** was supported by the upfield amidic carbonyl resonance at δ 164.2 instead of 173.0 ppm in **1**. In addition, IR band at 1655 cm^−1^ supported the presence of an amidic carbonyl in **3**. Again, the assignments of all quaternary carbons were secured from HMBC cross peaks (Table 2) completing the assignment of **3**.

Hyrtioerectines D–F (**1**–**3**) represent additional candidates of the rare alkaloids in which the indole and β-carboline fragments are linked through C-3 of both moieties. We have previously reported one compound of this series, hyrtioerectine A [12]. Additional member of this class including eudistomin U was reported from the Caribbean ascidian *Lissoclinum fragile* [20]. However, the linkage of the β-carboline and indole fragments in eudistomin U occurs between C-1 and C-3, respectively. In the case of hyrtioerectines, the linkage occurs between C-3 and C-3 of both moieties.

Compounds **1**–**3** were evaluated for their antimicrobial, free radical scavenging activity as well as cancer growth inhibition activity against three cancer cell lines. In the antimicrobial assay, compounds **1**–**3** displayed inhibition zones of 17, 9 and 14 mm against *Candida albicans* compared to 35 mm illustrated by clotrimazole at the same concentration. Furthermore, compounds **1**–**3** showed inhibition zones of 20, 10 and 16 mm against *Staphylococcus aureus*, respectively, compared to 30 mm illustrated by ampicillin. Finally, compounds **1**–**3** displayed weak inhibition zones of 7–9 mm against *Pseudomonas aeruginosa* compared to 30 mm illustrated by imipenem and were inactive against *E. coli*.

In the free radical scavenging activity assay using DPPH, compounds **1** and **3** were the most active compounds with 45% and 42% inhibition, respectively, while compound **2** were moderately active with 31% inhibition. Compounds **1**–**3** were evaluated for *in vitro* growth inhibition of lung carcinoma (A549), colorectal carcinoma (HT29), and breast adenocarcinoma (MDA-MB-231) cells (Table 3). Compounds **1**–**3** exhibited variable activity with GI_50_ values (the concentration required to achieve 50% growth inhibition of the cells) in the micromolar range and no selectivity between the cell lines tested. While compounds **1** and **3** were slightly more cytotoxic than compounds **2**, the compounds were more than 75–100 times less potent than the positive control doxorubicin (Table 3).

In conclusion, the relatively higher antimicrobial, free radical scavenging and growth inhibition activities of compounds **1** and **3** over **2** in could be attributed to the presence of diphenolic moieties in **1** and **3**. The amidation of the carboxylic moiety in **3** seems to have a slight effect on the activity if compared to **1**.

**Table 3:** Cancer cell line inhibition by hyrtioerectines D–F (**1**–**3**).

Compound	Cell line [GI_50_ (μM)]		
	MDA-MB-231	A549	HT-29
Compound **1**	25	30	28
Compound **2**	90	100	85
Compound **3**	42	35	45
Doxorubicin ^a^	0.30	0.35	0.40

^a^ Positive antiproliferative control.

## 3. Experimental Section

### 3.1. General Experimental Procedures

UV spectra were recorded on a Hitachi 300 spectrometer. IR spectra were recorded on a Perkin-Elmer 1310/84 Spectrometer. NMR spectra were obtained in CD_3_OD on Bruker Avance DRX 400 Spectrometers at 400 MHz for ^1^H NMR and 100 MHz for ^13^C NMR. NMR chemical shifts are expressed in parts per million (ppm) referenced to CD_3_OD solvent signals (δ 3.29 for ^1^H and δ 49.0 for ^13^C). ESI-MS spectral data were obtained with a Micromass Q-tof equipped with lockspray mass spectrometer using Leucine Enkaphalin at *m/z* 556.2771 [M + H]^+^ as a reference mass. For column chromatography, silica gel (Merck, 70-230 mesh ASTM) and Sephadex LH-20 (Pharmacia) were used. Pre-coated silica gel 60 F-254 plates (Merck) were used for TLC. DPPH was purchased from Sigma Aldrich, USA.

### 3.2. Animal Specimen

Specimen of the Red Sea sponge *Hyrtios* species (class Demospongiae, order Dictyoceratida, family Thorectidae) was collected from the Red Sea using SCUBA at depths between 10 and 18 m in 2011. The sponge was kindly identified by Dr. Rob van Soest. A voucher specimen was kept in our Red Sea Marine Invertebrates Collection under the registration number DY2011-49. The sponge forms pinkish-gray tubes, diameter of 4.5−5.0 cm, walls 0.5−1.0 cm thick. The inner lumen measures about 3.0 cm in diameter, with a smaller side tube of 2.0 cm diameter and lumen of 1.0 cm. The larger tube is about 8.0 cm high. Surface of the sponge is densely conulose, with blunt conules of 1.0 mm high, and 1.0−2.0 mm apart. The consistency is firm, crumbly, and sandy. The skeleton is dense, anisotropic, and consisting of sand-filled primary and secondary fibers, near the surface forming fascicles. Individual primary fibers measure 220−385 μm in diameter, and secondary fibers with diameter of 100−130 μm. Meshes polygonal or rounded with 250−1000 μm in diameter. Between the fibers the mesophyl is lightly charged with debris and sand grains. The surface aspect and skeletal characters conform to the genus *Hyrtios* (class Demospongiae, order Dictyoceratida, family Thorectidae), but there are no matching descriptions at the species level for this sponge. The voucher is kept in the collections of the Zoological Museum of the University of Amsterdam, under registration No. ZMA POR. 17249.

### 3.3. Extraction and Purifications of Compounds **1–3**

The lyophilized sponge materials (170 g) were extracted three times (3 × 500 mL) with a mixture of methanol/dichloromethane (1:1) at room temperature. The combined organic extracts were concentrated under reduced pressure and suspended in 400 mL of methanol/water (9:1). The resulted mixture was extracted with *n*-hexane (3 × 300 mL) to give 1.9 g of *n*-hexane extract. The remaining methanolic layer was diluted with water to (3:2) methanol/water and then extracted successively with dichloromethane (3 × 300 mL), ethyl acetate (3 × 300 mL) and finally with *n*-butanol (3 × 300 mL). Each of the extracts was concentrated under reduced pressure to give 1.2 g of CH_2_Cl_2_ extract, 530 mg of ethyl acetate extract and 3.8 g of *n*-butanol extract, respectively. All fractions were evaluated for antimicrobial and free radical scavenging activity. The ethyl acetate extract showed significant activity in both assays and thus was considered for further purification. The ethyl acetate residue (530 mg) was subjected to size exclusion chromatography on a Sephadex LH-20 column equilibrated with methanol to give five fractions. Fractions 3 and 4 were combined and were subjected to another Sephadex LH-20 column using methanol as an eluting solvent to give 6 subfractions. Subfraction 4 (39 mg) was purified on a semi-preparative C_18_ HPLC column using 20% acetonitrile in water at a flow rate of 2 mL/min to give compound **2** (4.3 mg). Subfraction 5 (33 mg) was subjected to final purification on a semi-preparative C_18_ HPLC column using 15% acetonitrile at a flow rate of 2 mL/min to afford compounds **1** (2.1 mg) and **3** (1.8 mg).

**Hyrtioerectine D** (**1**): yellow solid; UV (MeOH) λ_max_ (log ε) 387 nm (3.85), 248 nm (3.85), 312 nm (4.05); IR ν_max_ (film) 3600, 3500, 3020, 2980, 1725, 1610, 1405, 1290, 1255, 1220, 890 cm^−1^; NMR data, see Table 1, Table 2; positive HRESIMS *m/z* 360.0981 (calcd for C_20_H_14_N_3_O_4_ [M + H]^+^, 360.0984).

**Hyrtioerectine E** (**2**): yellow solid; UV (MeOH) λ_max_ (log ε) 420 nm (3.90), 252 nm (3.95), 315 nm (4.15); IR ν_max_ (film) 3595, 3545, 3015, 2980, 1720, 1612, 1410, 1290, 1250, 1220, 895 cm^−1^; NMR data, see Table 1, Table 2; positive HRESIMS *m/z* 374.1138 (calcd for C_21_H_16_N_3_O_4_ [M + H]^+^, 374.1140).

**Hyrtioerectine F** (**3**): yellow solid; UV (MeOH) λ_max_ (log ε) 380 nm (3.95), 240 nm (4.10), 315 nm (4.00); IR ν_max_ (film) 3590, 3500, 3350, 3010, 2985, 1655, 1615, 1405, 1290, 1255, 1215, 895 cm^−1^; NMR data, see Table 1, Table 2; positive HRESIMS *m/z* 359.1142 (calcd for C_20_H_15_N_4_O_3_ [M + H]^+^, 359.1144). 

### 3.4. Biological Evaluation of the Compounds

#### 3.4.1. Determination of the Antimicrobial Activity

The compounds were evaluated for their antimicrobial activity against a Gram positive bacterium (*Staphylococcus aureus* ATCC 6538), two Gram negative bacteria (*Escherichia coli* ATCC 8739), *Pseudomonas aeruginosa* ATCC 9027) and a yeast (*Candida albicans* ATCC 2091) using agar diffusion method. Accurately weighed 1 mg of test compound was dissolved in 1 mL DMF and 100 μL of the solution were inserted in the cups then incubated at 37 °C for 24 h. The inhibition zones were measured and compared with the reference antibiotics ampicillin (10 μg/disc), imipenem (10 μg/disc) or the antifungal drug clotrimazole (10 mg/mL). 

#### 3.4.2. Determination of the Free Radical Scavenging Activity Using DPPH

Compounds **1**–**3** were evaluated for their free radical scavenging activity using 2,2-diphenyl-1-picrylhydrazyl (DPPH). DPPH was prepared as 6 × 10^−5^ M solution in methanol, protected from light and kept in refrigerator. Aliquots of different concentrations from each compound were pipetted into a series of 5 mL volumetric flasks. To each flask 3 mL of DPPH solution were added, mixed with the solution, volumes were made up with methanol and flasks were allowed to stand in dark at room temperature for 10 min [21]. The absorbance of each of the resulted solutions was measured at 516 nm against similarly treated blank. The free radical scavenging activity was determined for each of the compound according to the following equation:

[% DPPH radical scavenging = (*A*_0_ − *A*/*A*_0_) × 100] (1)

where *A*_0_ = absorbance of a blank, *A* = absorbance of the sample.

#### 3.4.3. Cancer Cell Growth Inhibition Assay

Three cancer cell lines were used in this assay, namely lung carcinoma (A549, ATCC CCL-185), colorectal carcinoma (HT29, ATCC HTB-38), and breast adenocarcinoma cell (MDA-MB-231, ATCC HTB-26). The cancer cell lines were obtained from American Type Culture Collection (ATCC). The cell lines were maintained in RPMI medium supplemented with 10% fetal calf serum (FCS), 2 mM l-glutamine and 100 U/mL penicillin and streptomycin, at 37 °C and 5% CO_2_. Triplicate cultures were incubated for 72 h in the presence or absence of test compounds (at ten concentrations ranging from 10 to 0.0026 μg/mL). For quantitative estimation of cancer cell growth inhibition, the colorimetric sulforhodamine B (SRB) method was used [22]. Briefly, the cells were washed twice with PBS, fixed for 15 min in 1% glutaraldehyde solution, rinsed twice in PBS, and stained in 0.4% SRB solution for 30 min at room temperature. The cells were then rinsed several times with 1% acetic acid solution and air-dried. Sulforhodamine B was then extracted in 10 mM trizma base solution and the absorbance measured at 490 nm. Results are expressed as GI_50_, the concentration that causes 50% inhibition in cell growth after correction for cell count at the start of the experiment (NCI algorithm). Doxorubicin and DMSO (solvent) were used as the positive and negative controls in this assay. Prism 3.03 from GraphPad was used for the statistical analysis of the cell growth inhibition results.

## 4. Conclusions

In conclusion, our search for marine-derived bioactive compounds has led to the investigation of specimen of the Red Sea marine sponge *Hyrtios* species. Three new alkaloids, hyrtioerectines D–F (**1**–**3**), were isolated and their chemical structures were assigned using spectroscopic studies. The alkaloids belong to the rare class in which the indole and the β-carboline fragments are connected via C-3/C-3 of both moieties. Biological evaluation of the compounds showed that compounds **1**–**3** possess variable antimicrobial, free radical scavenging and cancer growth inhibition activities.
